# Patterns of ethical issues and decision-making challenges in clinical practice among Ghanaian physiotherapists

**DOI:** 10.4314/gmj.v54i3.9

**Published:** 2020-09

**Authors:** Gifty G Nyante, Caleb K Andoh, Ajediran I Bello

**Affiliations:** Department of Physiotherapy, School of Biomedical and Allied Health Sciences, College of Health Sciences, University of Ghana

**Keywords:** Ethical issues, code of ethics, decision making, physiotherapy practice, ethical judgement

## Abstract

**Objectives:**

To determine the patterns of ethical issues and decision-making challenges encountered by practicing physiotherapists in Ghana.

**Design:**

This is a cross-sectional study in which the stratified sampling technique was adopted to sample the participants.

**Setting:**

The study involved physiotherapists at the private healthcare setting and from different levels of public healthcare facilities.

**Participants:**

Eighty-two duly registered physiotherapists who were practising in Ghana participated in the study.

**Interventions:**

Participants completed a 30-item questionnaire related to ethical issues and challenges encountered in making ethical decisions. Data analysis was premised on the frequency of occurrence of ethical tensions and difficulty in decision making which were dichotomized as ‘high’ and ‘low’ issues, and ‘extreme’ and ‘low’ difficult decisions, respectively.

**Results:**

The age range of the participants was 21–49 years (mean 31.5 ± 1.4years). 18 (22%), 31 (37.8%) and 33 (40.2%) physiotherapists practice in the primary, secondary and tertiary healthcare settings respectively. 56 (68.3%) and 43 (52.4%) of the participants affirmed that ‘establishing priorities for patient's treatment amidst limited time resources’ was the most frequently encountered and the most extremely difficult ethical issue to make a decision on respectively. Whereas, limiting physical therapy services for personal or organizational gains sub-theme was the least occurred issue which was also the least difficult to make a decision on as indicated by the respective 16 (19.5%) and 18 (22.0%) physiotherapists.

**Conclusion:**

A wide range of primary and secondary ethical issues were reported by the sampled physiotherapists, which tend to pose difficulty during the decision-making process in practice.

**Funding:**

The research work was self-funded by the authors.

## Introduction

Ethics forms an integral component of healthcare practice which underscores the need to profile the patterns of practice-related ethical tensions in physiotherapy practice with specific reference to Ghana. Code of ethics remains a valid indicator for professional identity and provides a positive framework for therapists' personal conduct, their relationship with the patients and other health care team members.^1^ Regulations or laws set the pace for professional behaviour whilst codes of ethics set goals for professionalism. All healthcare professionals through training are bound by specific frameworks of ethics as dictated by their peculiar approaches.

Physiotherapy practice entails applications of various manual techniques and therapeutic appliances towards ensuring optimal movement and quality of life.^2^ To meet the society's mutable expectations therefore, physiotherapy practice demands continuous development and ethical competences of its practitioners. Physiotherapy services are aimed at beneficence (duty to help), non-maleficence (duty to avoid harm) and respect for patients' autonomy (patient's “right to hold views, make choices and take actions based on personal values and beliefs”).^3^

Although code of ethics is well reported in the literature for healthcare practices including physiotherapy, there is always an attempt to generalize its applicability to all practice environments despite the possible divergent reactions from practitioners due to their values and beliefs. The primary target of any healthcare ethics is to improve the quality of care delivery by delineating ethical issues that arise in practice to resolve them.

Consideration of the specific practice environment is therefore necessary concerning the ethical tensions and decision making as parts of the clinical judgment components. Even though the practice of physiotherapy began as an adjunct to medical practice including absolute dependency on the clinical and ethical decisions of the referring physicians, the rising trend of professional autonomy has elicited its peculiar ethical dilemmas within the profession.^4^ Ethical issues or dilemmas often occur when any two of the major ethics principles are conflicting thereby underscoring ethical competence to make decisions.

This is most common when there is an occurrence of mismatch between the requisite professional approaches and beliefs and/or moral values of the healthcare consumers. Nortje, and de Jongh, grouped the ethical tensions into three themes as; competence and conduct such as abandonment and insufficient time, business practices regarding contractual obligations resulting in fraudulent billing as well as a professional practice associated with overfamiliarity.^5^

Ethical tensions and the associated decisions are crucial in physiotherapy practice given the close therapist-patient interaction, relationships with other healthcare providers, informal caregiver's demands and the utilization of therapeutic devices. For instance, an Author provided four key sources of ethical tensions that arose from the manufacture and utilization of therapeutic devices in physiotherapy practice to include bioengineering in physical therapy, ethical and clinical standards for manufacturers; social impact of physical therapy devices and ethical issues; inter-professional lack of communication and ethical concerns; bioengineering ethical research and education.^6^

Similarly, Rendeau et al, reported ethical issues experienced by Athletic Therapists and Physical Therapists with university sports team as arising from maintaining professional boundaries among the healthcare team (e.g. team communication), striving for respectful and effective collaboration (e.g. interdisciplinary decision making), frequently seeking answers to ethical issues (e.g. athlete's personal and health issues) and living with the repercussions of challenging decisions (e.g. confidentiality and privacy).^7^ Pattern of responses to ethical dilemma in practice among physiotherapists is not yet documented in Ghana in spite of the possible influence of culture and practice environment. The on-going professional expansion in healthcare service has also challenged Physiotherapists to adhere to all social etiquettes associated with their practice on a daily basis.^1^ Ethics knowledge and practice in physiotherapy continues to suggest further identification and examination of ethical issues as an important activity and hallmark of professionalism. Thus, it is relevant to ascertain ethical issues that arise in the course of physiotherapy sessions in Ghana and the challenges encountered in making decisions.

## Methods

### Participants

Practising physiotherapists, who were resident in Ghana, participated in this study. They were included to take part in the cross-sectional survey if they were licensed by Allied Health Professions Council-Ghana and were duly registered as members with Ghana Physiotherapy Association (GPA). Physiotherapists who were academics without regular contact with patients and those who have retired from active practice were excluded from the study. Participants were sampled through stratified random sampling technique from private, public secondary and tertiary healthcare settings as obtained from the GPA registry.

Proportional sampling quota was employed to sample participants from each group according to the population sizes. The eventual study sample size n was estimated from Slovin's formula:^8^ n=N/1+Ne in which N is the population size, which was 95 registered members as at the time of the conduct of this study and e (0.05) is taken as a margin of error. Thus, a minimum of 77 registered physiotherapists was required to participate in the study. Invariably, the number was increased to 82 (86.3%) to manage defaulting participants.

### Instruments for data collection

Information on age, sex, years of physiotherapists' practice experience, education qualification and respondents' present employment facilities were captured in a short data form. A 30-item questionnaire which consists of six domains with various practice-related ethical issues was used to collect data.^9^ Responses on each ethical issue is rated on five-point Likert scale as ‘high’, ‘moderate’, ‘minimal’, ‘none’ and ‘not applicable’ whilst responses on decision making on each issue is rated on four-point scales as ‘extreme’ ‘moderate’ ‘minimal’ and ‘none’.

### Procedure for data collection

The Ethics and Protocol Review Committee of School of Biomedical and Allied Health Sciences approved the study protocol (Ethics Identification Number: SBAHSET./10381992/AA/11A/2012-2013).

Upon obtaining permission from the GPA secretariat to carry out the study on her members, the lists of the physiotherapists and their healthcare facilities were obtained ahead of the GPA annual general meeting. The list was used to randomly sample the participants based on the status of their health facilities and the available number of physiotherapists during their meeting. An information sheet detailing the aim and methodology of the study was provided for all the participants.

A written informed consent form was also made available through which those who agreed to participate in the study gave approval. Copies of the self-administered questionnaire were thereafter distributed. Participants were required to rate ethics-related issues according to the frequency of occurrence in their practice as well as the difficulty they faced in making decisions on such tensions. In the event of no encounter with a particular ethical issue as outlined in the questionnaire, rating of the decision on such issue would not also be necessary. Each participant was given ample time of at least two days to return the questionnaire. Given the interest generated by the study among the members, researchers were able to retrieve all the copies of the administered questionnaires.

### Data Analysis

Data analysis was performed with SPSS statistical software package version 16. The social-demographic variables such as sex, age, education status, duration of practice and years of experience were presented with descriptive statistics such as means, standard deviation, frequency and percentage. The ethical issues encountered and decisions made by the respondents were also presented descriptively with frequency and percentage.

Responses on the frequency of occurrences of ethical issues were dichotomized as ‘high’ (comprising ‘high’ and ‘moderate’ response options) and ‘low’ (comprising ‘minimal’, ‘none’ and ‘not applicable’ response options). Similarly, responses on the difficulty in decision making were also dichotomized as ‘Extremely difficult’ (comprising ‘extremely difficult’ and ‘moderately difficult’ decision making) and ‘least difficult’ (comprising ‘minimally difficult’ and ‘not difficult’ decision-making options) on the Likert scale for the ease of statistical presentation.

Ethical issues are also classified as primary and secondary tensions. Primary ethical tensions are suggestive of those that occur most frequently coupled with difficulty in decision making as alluded to by ≥ 40% of the respondents.^9^ Secondary issues are those that meet either the high frequency of occurrence or extremely difficult decision criterion but not both, whereas tertiary tensions are those that do not meet both criteria levels as alluded to by ≥ 40% of the participants.

## Results

### Socio-demographic profiles of the participants

**Figure 1:**
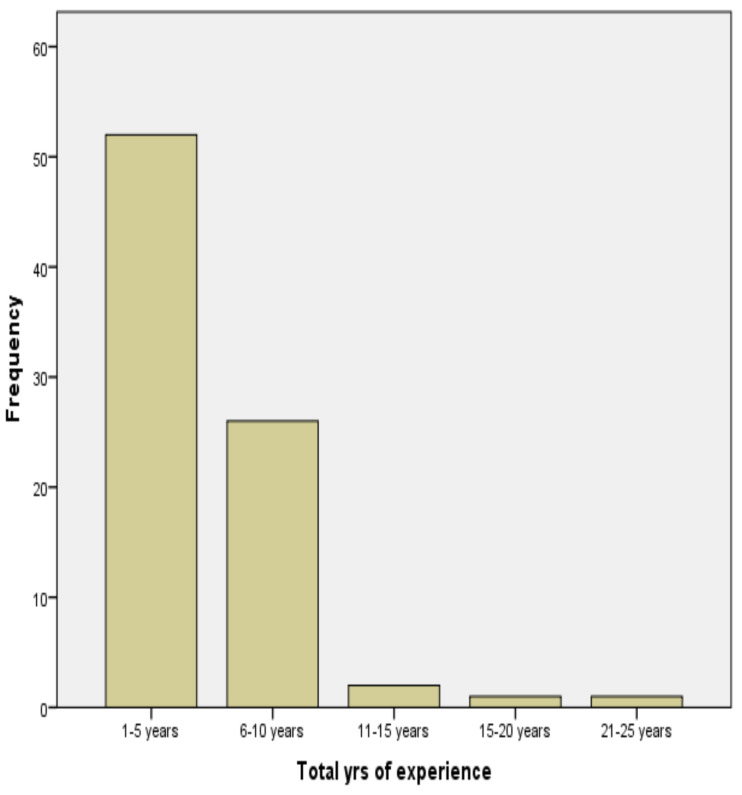
Years of practice experience among the physiotherapists.

Eighty-Two physiotherapists took part in this study, of which 46 (56.1%) were males, and 36 43.9% were females. The mean age of the participants was 31.5 ± 1.4years (age range 21–49 years) Table 1. Regarding the health facility status, 33 (40.2%) participants were practising in public tertiary healthcare setting compared to 31 (37.8%) in secondary setting and 18 (22.0%) in private care practice. A total of 74 (90.2%) physiotherapists practised with a Bachelor of Science degree in physiotherapy compared with 8 (9.8%) participants who had received postgraduate training. Almost two-thirds of the participants, 52 (63.4%) had less than 6-years practice experience whilst only 2 (2.4%) participants had practised for over 15 years (Figure 1).

**Table 1:** Socio-demographic profiles of the participants

Variable	n (%)
**Gender**	
**Male**	46 (56.1)
**Female**	36 (43.9)
**Total**	82 (100)
**Age group**	
**21 to 25 years**	15 (18.3)
**26 to 30 years**	41 (50.0)
**31 to 35 years**	17 (20.7)
**36 to 40 years**	4 (4.9)
**41 to 45 years**	2 (2.4)
**46 to 50 years**	3 (3.7)
**Total**	82 (100)

### Participants' responses on the frequency at which each ethical issue was encountered and the level of difficulty faced in making decision

Out of the six domains, the most frequently encountered ethical tension and the most extremely difficult issue to make decision on was recorded on the sub-theme: “establishing priorities for patient treatment when time or resources are limited” as reported by 56 (68.3%) and 43 (52.4%) physiotherapists respectively. This was reported under the domain: ‘decisions regarding the choice to treat’.

The highest frequency of encounters with ethical issues were recorded in the domain of ‘obligations deriving from the patient-therapist contract’ in which six out of the seven sub-themes were reported by the participants as high. On the other hand, ‘withholding or limiting physical therapy services in order to improve work conditions, salaries, staff/patient ratios was indicated as the lowest ethical issue and the least difficult to decide on as reported by 16(19.5%) and 18(22.0%) participants, respectively.

In all, four primary ethical issues were indicated by the participants across the domains of the professional ethics: establishing priorities for patient treatment when time or resources are limited, 56(68.3%) and 43(52.4%); determining professional responsibilities when a patient's needs or goals conflict with the family's needs or goals, 37(45.1%) and 39(40.2%); deciding whether to represent certain necessary patient services in a way that would meet third party-payer limitations, 45(54.9%) and 34(41.4%); accepting gratuities or gifts from patients/families, 45(54.8%) and 34(41.5%). In addition, majority of the secondary ethical issues encountered were reported in the domain of ‘obligations derived from the patienttherapist contract’, as alluded to by ≥40 of the participants either as being frequently encountered or difficult to make a decision on. The results are presented in Table 2.

**Table 2:** Ethical issues and difficult decision-making process

Ethical issues	Responses on the frequency of encounter: n (%)		Responses on the difficulty in decision making: n (%)	
**Decisions regarding the choice to treat**	High	Low	Extreme	Low
Sub-themes				
**1. Establishing priorities for patient treatment when time or resources are limited**	56(68.3)	26(31.7	43(52.4)	39(47.6)
**2. Discontinuing treatment for patients who habitually disregard instructions such as for home programs,** **treatment regimens, and safety instructions**	20(24.4)	62 (75.6)	35(42.7)	47(57.3)
**3. Continuing treatment with a terminally ill patient**	44(53.7)	38(46.3)	32(39.0)	50(61.0)
**4. Continuing treatment to provide psychological support after physical therapy treatment goals have** **been reached**	9(47.6)	43(52.4)	29(35.4)	53(64.6)
**Obligations deriving from the patient-therapist contract**				
**5. Determining professional responsibilities when a patient's needs or goals conflict with the family's** **needs or goals.**	37(45.1)	45(54.9)	39(40.2)	43(59.8)
**6. Defining the limits of the physical therapist's role in the initial education of a patient/family regarding** **diagnosis or prognosis**	68(82.9)	14(17.1)	16(19.5)	66(80.5)
**7. Informing a patient/family about the limitations of treatment**	72(87.8)	10(12.2)	24(29.3)	58(70.7)
**8. Assuring that the patient/family has input into treatment and discharge planning.**	78(95.1)	14 (4.9)	15(18.3)	67(81.7)
**9. Assuming personal responsibility for continuing education to keep up with new treatment ideas in** **order to maintain quality of care.**	75(91.5)	7 (8.5)	31(37.8)	51(62.2)
**10. Weighing the effects of treatment against the discomfort created by the procedure**	60(73.2)	22(26.8)	29(35.4)	53(64.6)
**11. Maintaining a patient's sense of personal space and dignity when treatment requires arrangements** **such as close proximity and group settings.**	65(79.3)	17(20.7)	21(25.6)	61(74.4)
**Moral obligation and economic issues**				
**Sub-themes**				
**12. Deciding whether to represent certain necessary patient services in a way that would meet third** **party-payer limitations.**	45(54.9)	37(45.1)	34(41.4)	48(58.6)
**13. Withholding or limiting physical therapy services in order to improve work conditions, salaries,** **staff/patient ratios**	16(19.5)	66(80.5)	18(22.0)	64(78.0)
**Sub-themes**				
**Physical Therapist's relationship to other health professionals**				
**Sub-themes**				
**14. Maintaining a patient's/family's confidence in other health professionals regardless of personal** **opinions**	67(81.7)	15(18.3)	18(22.0)	64(78.0)
**15. Determining criteria for delegating duties to supportive personnel**	68(82.9)	14(17.1)	18(22.0)	64(78.0)
**16. Reporting questionable practices of another physical therapist to the appropriate person.**	29(35.4)	53(64.6)	32(39.0)	50(61.0)
**17. Reporting questionable practices of a physician to the appropriate person**	26(31.7)	56(68.3)	29(35.4)	53(64.6)
**18. Reporting questionable practices of another health professional who is not a physical therapist or** **a physician to the appropriate person.**	32(39.0)	50(61.0)	22(26.8)	60(73.2)
**Conflicts between two ethical principles**				
**Sub-theme**				
**19. Deciding what to do when two of my ethical principles or values are in conflict**	20(24.4)	62 (75.6)	36 (43.9)	48(56.1)
**Sub-themes**	High	Low	Extreme	Low
**20. Deciding criteria for allowing a patient/ family to refuse treatment.**	23(28.0)	59 (72.0)	31(37.8)	51 (62.2)
**21. Accepting gratuities or gifts from patients/ families**	45(54.8)	37 (45.2)	34(41.5)	48 (58.5)
**22. Deciding what to do when my values and beliefs are at odds with a patient's/family's values and** **beliefs.**	27(32.9)	55 (67.1)	19(23.2)	63 (76.8)
**23. Setting the limits necessary to maintain professional relationships with patients/ families.**	66(80.4)	16 (19.6)	14(17.1)	68(82.9)
**24. Controlling access to privileged or confidential information about a patient/ family**	67(81.7)	15 (18.3)	14(17.1)	68(82.9)
**25. Choosing a form of dress for work that assures professional respect and maintains identity as a** **physical therapist.**	55(67.1)	27(32.9)	6(7.3)	76(92.7)
**26. Deciding when I do not have adequate therapeutic knowledge to treat a patient.**	18(22.0)	64 (78.0)	11(13.4)	71(86.6)
**27. Setting financially sound fees that maintain a patient's ability to receive treatment.**	45(54.9)	37(45.1)	18(22.0)	64(78.0)
**28. Providing accurate information to consumers about the costs of treatment**	73(89.0)	9 (11.0)	11(13.4)	71(86.6)
**29. Determining methods for making the particulars of physical therapy services known to health** **care consumers.**	59(72.0)	23(28.0)	15(18.3)	67(81.7)
**30. Deciding the limits for standing by my own ethical principles**	58(70.7)	24(29.3)	19(23.2)	63(76.8)

## Discussion

The study was aimed at highlighting the patterns of ethical issues encountered by Ghanaian physiotherapists in their practice and the levels of difficulty they face in making ethical decisions. Four ethical issues were identified by the participants as primary issues of professional ethics as follows: 1. establishing priorities for patient treatment when time and resources are limited; 2. determining professional responsibilities when a patient's needs or goals conflict with the family's needs or goals; 3. decision on whether to represent certain necessary patient services in a way that would meet third party-payer limitations; 4. issue of accepting gratuities or gifts from patients/family.

Also, most ethical issues encountered were largely secondary and the majority of the participants indicated the ‘obligations deriving from the patient-therapist contract’ as the most reported theme in that regard. The reported ethical patterns in this study somewhat reflect the profile of the physiotherapists at the time of conducting this study.

It was found that 52 (63.4%) had less than 6-years practice experience, whilst only 2 (2.4%) participants had practised for over 15 years. This finding shows a relatively young group of health care professionals (31.5 ± 1.4years) who earnestly need guidance from the experienced practitioners as role models.

The relatively low profile clearly underscores mentorship in physiotherapy practice in Ghana to be able to incorporate adequate ethical practice. According to Burges and Jelsma, ethics of care stipulates that junior physiotherapists need to pay attention to the health care recipients in order to be sensitive to them within their moral, cultural and social contexts whilst young physiotherapists should in turn be supported and cared for by their senior colleagues as well as the rest of the multidisciplinary team members.^10^ Again, of all the participants, only 22% practice in a private healthcare setting compared to 37.8% and 40.2% who ply their trades in the secondary and tertiary health care settings respectively. It could thus be deduced that the patterns of presentation are largely tilted towards public health care setting.

### Practice-related ethics on decisions regarding the choice to carry out treatment

The ethical issue regarding ‘establishing priorities for patient's treatment when time or resources are limited’ was indicated as the most frequently occurred primary ethical issues and the most difficult to decide on. This suggests that Ghanaian physiotherapists are frequently confronted by overwhelming demands for treatment in the midst of constraints imposed by time and material resources. This finding can be deciphered in two ways. On the one hand, the high physiotherapists-residents ratio in Ghana which stood at 1: 1,910, 441 in 2017 is grossly inadequate to provide rehabilitation services to the teeming population of clients.^11^

This short fall raises concerns about the capability of the available physiotherapists to cope with the rehabilitation needs of the clients thereby potentiating ethical tensions and challenges to make decisions in the event of ethical issues arising among the few available professionals. On the other hand, this finding could also be attributed to limited material resources such as space and limited physiotherapy facilities needed for such demands, thereby undermining their rehabilitation service delivery. Suffice it to state that several challenges still plague the health care systems in developing countries. For instance, most participants (78%) practice in public health facilities, leaving 22% in private practice.

It thus suggests that private health facilities that provide rehabilitation services are very scanty. Globally, the adoption of privatization seems to be on the rise and the private sector is known to play a critical role in the provision of health care services. However, the high costs of pharmaceuticals and health-related equipment are often beyond the reach of the intending health care investors in developing countries including Ghana.

This often contributes to the high cost of health care delivery thus posing as barrier to health care accessibility. In Africa, the Government annual budget allocation for health care is usually sub-optimal thus making health care unduly expensive. Indeed, the out-of-pocket expenses accounted for a whopping 67% of the total health care expenses in Ghana as contained in the World Bank report in 2017, compared to 13% in South Africa, and 21% in the USA.^12^ These challenges could only elicit ethical tensions in the course of health care provision for the clients.

### Obligations deriving from the patient-therapist contract

This theme consists of seven sub-themes of which six were secondary ethical issues and the sub-theme on ‘determining professional responsibilities when a patient's needs or goals conflict with the family's needs or goals issues’ was rated a primary ethical issue by ≥40% of the participants. This domain represents the most critical ethical issues experienced by the participants in this study either with regard to the frequency of encounter or the bane of decision making. This finding is not surprising given the cross-cultural situation in which the participants were practicing.

Since close interaction exist between physiotherapists and their patients, understanding culture in the context of how to choose, access, accept and respond to health care is an essential component of clinical judgment for health care professionals including physiotherapists.^13^ Indeed, acquisition of such skill is interpreted in the realm of cultural competence. For physiotherapists to be culturally competent, they must take into cognizance; their own cultural identity, possess cultural knowledge of common health beliefs and behaviours, display culturally sensitive behaviours and use these knowledge and skills to modify approach to be able to meet the diverse demands of their clients. 14, 15

### Moral obligation and economic issues

The sub-theme on the decision whether to represent certain necessary patient services in a way that would meet third party-payer limitations was also rated as primary ethical issues among the participants. Although health insurance scheme is being introduced into health sector in Ghana, physiotherapy is yet to be fully incorporated, which could be speculated to account for this finding. Our finding was similar to the report documented by Guccione.^9^

Poulis described 3 distinctive ethical issues that could emerge from clinical physical therapist practice as; 1) inherent goals and ideals of the physical therapy treatment encountered, 2) the interdisciplinary nature of the clinical environment that requires decisions to be made about the best interests of patients within a web of health professional teams, institutional and health policies, and 3) patient/family cultures and relationships.^16^ Participants in this study might have found themselves in the similar situation.

### Other Ethical issues

Participants also rated the sub-theme ‘accepting gratuities or gifts from patients/ families, as primary issue. The average age of the participants in this study was 31.5 ± 1.4years and 63.4% of them had only practiced less than six years. The relatively short practice experiences of the majority of the physiotherapists may be a key factor culminating in the lack of ethical competency which in turn could have elicited this ethical tension. Ethical judgement plays an increasingly important role in the clinical decisions of physiotherapists. As advocated by Bello and Adegoke, there is an urgent need to infuse ethical theory and skills into their daily decision-making processes.^1^ Ethics training creates room for critical thinking, objective analysis and clinical reasoning processes to inform decision making in different contexts and patients' backgrounds which should be free of bias.^15^, ^5^

## Conclusion

Findings from this study indicate that Ghanaian physiotherapists encountered a wide range of primary and secondary ethical issues across all aspects of professional health care ethics in their practice. They also experienced extreme difficulty to make decisions in the wake of such tensions. These findings underscore more training for ethical competency among the physiotherapists to inform their decision-making process in practice.

## Additional information

This paper was presented at the 6th International Conference on Ethics Education (6IEAA) on October 4, 2018, at Stellenbosch, Republic of South Africa.
